# Mechanical Circulatory Support for Advanced Heart Failure: Are We about to Witness a New “Gold Standard”?

**DOI:** 10.3390/jcdd3040035

**Published:** 2016-12-12

**Authors:** Massimo Capoccia

**Affiliations:** 1 Scottish National Advanced Heart Failure Service, Golden Jubilee National Hospital, Glasgow G81 4DY, UK; capoccia@doctors.org.uk; Tel.: 44-141-9515000; 2 Biomedical Engineering, University of Strathclyde, Glasgow G4 0NW, UK

**Keywords:** LVAD, rotary blood pumps, patient-specific modelling

## Abstract

The impact of left ventricular assist devices (LVADs) for the treatment of advanced heart failure has played a significant role as a bridge to transplant and more recently as a long-term solution for non-eligible candidates. Continuous flow left ventricular assist devices (CF-LVADs), based on axial and centrifugal design, are currently the most popular devices in view of their smaller size, increased reliability and higher durability compared to pulsatile flow left ventricular assist devices (PF-LVADs). The trend towards their use is increasing. Therefore, it has become mandatory to understand the physics and the mathematics behind their mode of operation for appropriate device selection and simulation set up. For this purpose, this review covers some of these aspects. Although very successful and technologically advanced, they have been associated with complications such as pump thrombosis, haemolysis, aortic regurgitation, gastro-intestinal bleeding and arterio-venous malformations. There is perception that the reduced arterial pulsatility may be responsible for these complications. A flow modulation control approach is currently being investigated in order to generate pulsatility in rotary blood pumps. Thrombus formation remains the most feared complication that can affect clinical outcome. The development of a preoperative strategy aimed at the reduction of complications and patient-device suitability may be appropriate. Patient-specific modelling based on 3D reconstruction from CT-scan combined with computational fluid dynamic studies is an attractive solution in order to identify potential areas of stagnation or challenging anatomy that could be addressed to achieve the desired outcome. The HeartMate II (axial) and the HeartWare HVAD (centrifugal) rotary blood pumps have been now used worldwide with proven outcome. The HeartMate III (centrifugal) is now emerging as the new promising device with encouraging preliminary results. There are now enough pumps on the market: it is time to focus on the complications in order to achieve the full potential and selling-point of this type of technology for the treatment of the increasing heart failure patient population.

## 1. Introduction

Whether we like it or not, a biological solution remains inadequate to address the magnitude and severity of advanced heart failure. There is overwhelming evidence to support the use of rotary blood pumps for the treatment of advanced heart failure either as a bridge to transplant or as a more permanent solution for non-eligible candidates. Since the REMATCH Trial [1,2], the technological development from pulsatile to continuous flow ventricular assist devices has led to an increased survival of patients on prolonged circulatory support [3,4,5,6]. CF-LVADs have proven their reliability with favourable outcome following elective surgery in chronic heart failure patients before the onset of cardiogenic shock [5]. In patients up to 70 years of age without cardiogenic shock, diabetes and renal failure, circulatory support with CF-LVADs showed 1- and 2-year survival of 80% and 70%, which was comparable with heart transplantation [6,7]. A recent analysis of the Interagency Registry for Mechanically Assisted Circulatory Support (INTERMACS) has showed greater durability for continuous flow LVADs in comparison with pulsatile flow devices [8] and confirmed its increasing trend [9]. Durability issues are closely related to driveline infection and failure, thrombosis and haemolysis, and ultimately pump drive unit failure.

The decision to insert a device is complex and must take into account the experience of the surgical team, potential risks and complications and the latest data on outcomes with newer devices. There are a number of devices currently available on the market and under development but only a few have met specific requirements to be introduced in clinical practice. Indication, timing and selection of the appropriate device are very important. Additional issues include quality of life, reliability and cost-effectiveness. From an engineering point of view, the greatest challenge is the development of a permanent circulatory support system, which is anatomically adaptable, highly resistant to operate in a corrosive saline environment and structurally compatible to be joined to the flexible tissues of the body. Such systems are expected to operate continuously for years without maintenance. Mechanical components are necessarily small and should operate without failing under extreme conditions. These devices should be efficient to reduce power requirement, prolong battery life and reduce waste heat. Blood trauma and flow disturbance should be within clinically acceptable limits. Blood-contacting surfaces should minimize potential generation of blood clots leading to cerebro-vascular accidents.

The purpose of this review is to discuss the concepts behind this type of technology with some considerations about current research with potential developments.

## 2. Type of Devices

Broadly speaking, there are two main categories of mechanical blood pumps: volume-displacement and rotary pumps.

Volume-displacement pumps are known as first generation devices. Their performance is excellent in unloading the left ventricle and sustaining the circulation with a capacity to pump up to 10 L/min of pulsatile flow. However, there are clear disadvantages such as large size, complexity, noisy operating mode and limited durability because of many moving parts [10,11].

Rotary pumps with continuous axial flow requiring mechanical bearings and seals in contact with blood are known as second generation devices. They are smaller and safer to insert with more favourable durability because of only one moving part. Thrombus formation remains a feared complication and varies between pumps. Experience with this type of devices is well established [4,12,13,14].

Rotary pumps with continuous flow based on magnetic levitation or non-contacting hydrodynamic bearings allowing the impeller to be suspended are known as third generation devices. These pumps are based on the concept of centrifugal flow and are even smaller than axial flow pumps. The use of magnetically levitated rotor systems is likely to improve durability. HeartWare, DuraHeart and HeartMate III are emerging devices based on these principles. Although clinical experience with these pumps is just getting under way, early results are promising [15,16,17,18,19].

## 3. Volume Displacement vs. Rotary Blood Pumps

Volume-displacement pumps consist of a chamber or a sac that fills passively or by suction and is compressed by an external pusher plate. Energy is transferred to the blood by periodic changes in a working space generating pulsatile flow. Inflow and outflow prosthetic valves are required. Devices such as HeartMate I XVE and Novacor are based on this principle.

The output requirement for a pulsatile configuration is a flow rate of 5–10 L/min at a mean pressure of 100–150 mmHg and a rate less than 120 bpm with a mean filling pressure of about 20 mmHg [20]. The human heart can be considered as a sophisticated volume displacement pump where the muscle contracts generating pressure work according to the following formula:

(1) $$dW=P\frac{dV}{dt}$$

Rotary blood pumps consist of an inlet and outlet with a single rotating element (impeller) that transfers energy to the blood in order to increase arterial blood flow and pressure. These devices can be axial, radial (centrifugal) and diagonal (mixed flow) according to the geometry of the impeller. Energy is transferred to the blood by velocity changes within the impeller vanes generating non-pulsatile flow. The mechanism of the rotating element in an axial-flow pump can be viewed as a screw-driving manoeuvre along the *x*-axis. In contrast, the rotating element in a centrifugal-flow pump has a helicopter-like behaviour along the *y*-axis.

Axial flow pumps (HeartMate II and Jarvik 2000) are driven by a spinning rotor around a central shaft. Centrifugal flow pumps (HeartWare, DuraHeart, HeartMate III) are driven by a hydrodynamic or electromagnetic suspended spinning rotor. Rotary blood pumps are suitable for high flows up to 20 L/min at differential pressures lower than 500 mmHg. The radial design is capable of producing high pressures and low flows. An axial flow pump generates high flows at low pressure differences. A diagonal pump is a mixed flow system capable of generating high pressures and high flows. Pump design is normalized to pump size taking into account that a 60-mm-diameter centrifugal pump can eject more fluid at significantly higher pressures than a 6-mm-diameter axial pump [21]. An increased resistance in rotary blood pumps leads to decreased work and therefore available power [22].

Instead, volume displacement pumps are suitable to generate pressure against resistance. They maintain a constant flow against an increasing resistance by generating a greater pressure at the expense of increased work and energy consumption. In the presence of very high resistance or flow stop, the pump fails. The heart is a modified but more complex volume displacement pump, which generates variable output according to preload, afterload and contractility. In the presence of very high afterload, failure is due to progressive endocardial ischaemia shown by the descending limb of the Frank-Starling curve.

Rotary blood pumps generate flow according to the amount of pressure dependent on resistance to flow. If the outflow graft of the HeartMate II (axial) or the HeatWare HVAD (centrifugal) is clamped, the true electrical and mechanical work done by the pump decreases although in theory the impeller may be pushing against an infinite resistance. In a clinical setting this would be paradoxical because myocardial oxygen demand and wall stress increase dramatically following aortic cross-clamp. The impeller rotates at high speed inside the pump housing with either forward (axial flow pump) or outward (centrifugal pump) fluid acceleration. The impeller’s ability to generate flow depends on the rotational speed, blade radius, blade pitch and blade height as described by Euler’s pump theory.

A combination of torque and velocity allow the impeller to transfer energy to the blood and generate flow and pressure. The rate at which a pump adds energy to a fluid is:

(2) $$\dot{W}=\frac{dV}{dt}\;\times \;\Delta P=Q\;\times \;\Delta P$$

where $Q=\;\frac{dV}{dt}$ is the flow and ∆*P* is the pressure.

The efficiency of a pump is defined as the ratio of the useful power output to the required power input:

(3) $$\eta_{pump}=\frac{Q\;\times \;\Delta P}{{\dot{W}}_{input}}$$

If the rotational speed ω and the torque T are known, then:

(4) $${\dot{W}}_{input}=T\;\times \;\omega$$

Therefore:

(5) $$\eta_{pump}=\frac{Q\;\times \;\Delta P}{T\;\times \;\omega }$$

But: ΔP = $\rho gh_{pump}$, where $h_{pump}$ is the pump head.

Therefore, the pump efficiency can be formulated as follows:

(6) $$\eta_{pump}=\frac{\rho Qgh_{pump}}{T\;\times \;\omega }$$

When the fluid thrust force does not act through the centre of gravity of a system, it generates a torque which produces rotational motion. The angular momentum will be the product of the length of a lever arm from a spatial coordinate origin times the linear momentum, which is the product of mass times velocity. In vector terms, the following expression [23] is obtained:

(7) $$\vec{H}=\vec{r}\times (m\times \vec{V})$$

where $\vec{H}$ is the vector of the angular momentum.

For a fluid system defined within a control volume, *V*, the total angular momentum is as follows:

(8) $$\vec{H}=\int_{V}\rho (\vec{r}\times \vec{V)}$$

The rate of change of the angular momentum of a fluid system equals the rate of change of fluid angular momentum within the control volume plus any changes in relation to convective fluxes of angular momentum in or out of the control volume:

(9) $$\frac{d\vec{H}}{dt}=\frac{\partial }{\partial t}\int_{V}\rho \left(\vec{r}\times \;\vec{V}\right)+\int_{S}\rho \left(\vec{r}\times \vec{V}\right)(\vec{V}\;•\;d\vec{A})$$

A torque produces a change in angular momentum. If we consider a right-hand coordinate system defined about an origin, O, the sum of any external torques on the system equals the total change in angular momentum according to the conservation law [23]. Therefore, the following general expression is obtained:

(10) $$\sum {\vec{M}}_{0}=\frac{d\vec{H}}{dt}=\frac{\partial }{\partial t}\int_{V}\rho \left(\vec{r}\times \;\vec{V}\right)+\int_{S}\rho \left(\vec{r}\times \vec{V}\right)(\vec{V}\;•\;d\vec{A})$$

In conditions of steady-state flow with uniform velocity profiles at inlet and outlet, the equation becomes:

(11) $$\sum {\vec{M}}_{0}=\sum {\dot{m}}_{\mathrm{out}}\left(\vec{r}\times \vec{V}\right)-\sum {\dot{m}}_{\mathrm{in}}\left(\vec{r}\times \vec{V}\right)$$

Newton’s second law states that the torque on the impeller is equal to the rate of change of the angular momentum of fluid.

Therefore:

(12) $$T=m\left(V_{u2}\times r_{2}-\;V_{u1}\times r_{1}\right)$$

According to Euler’s velocity triangles, the absolute fluid velocity is the sum of the relative velocity of fluid with respect to the moving blade and the blade velocity [24].

The pump power (${\dot{W}}_{pump}$) is the product of torque (T) and rotational speed (ω).

Therefore:

(13) $${\dot{W}}_{pump}=\omega \times T=m\left(V_{u2}\times \omega \times r_{2}-\;V_{u1}\times \omega \times r_{1}\right)=\;m\left(V_{u2}U_{2}-\;V_{u1}U_{1}\right)$$

where: $U_{1}$ = $\omega \times r_{1}$ blade speed at the inlet; $U_{2}$ = $\omega \times r_{2}$ blade speed at the outlet.

Dividing by mg, we obtain:

(14) $$\frac{{\dot{W}}_{pump}}{mg}=\frac{1}{g}(V_{u2}U_{2}-\;V_{u1}U_{1})$$

But: $h_{pump}$ = $\frac{{\dot{W}}_{pump}}{mg}$.

Therefore:

(15) $$h_{pump}=\frac{1}{g}(V_{u2}U_{2}-\;V_{u1}U_{1})$$

or

(16) $$g\cdot h_{pump}=(V_{u2}U_{2}-\;V_{u1}U_{1})$$

Then, the efficiency of the pump can be written in a more generalized form:

(17) $$\eta_{pump}=\frac{\rho Q}{T\omega }(V_{u2}U_{2}-\;V_{u1}U_{1})$$

According to Bernoulli’s equation, the total fluid energy at point A is the sum of pressure energy, kinetic energy and potential energy that must be preserved at point B as follows:

(18) $$P_{a}+\;\frac{1}{2}\;\rho v_{a}^{2}+\;\rho gh_{a}=\;P_{b}+\;\frac{1}{2}\;\rho v_{b}^{2}+\;\rho gh_{b}$$

The fluid is accelerated by the impeller’s rotation with the addition of mechanical energy to the kinetic component of flow. At the exit, the fluid decelerates shifting the added energy of flow back to pressure. This event depends on outflow resistance. At the inlet, suction occurs and power is consumed by the pump to produce hydraulic work and to overcome viscous friction. In the presence of an outflow obstruction leading to increased afterload in a rotary blood pump, the flow decreases and less work is done. The rotor spins at the same speed but the impeller contacts and thrusts less fluid and, as a consequence, does less work with a decrease in energy demand. Therefore, clamping the outflow graft of a rotary blood pump leaves the impeller rotating in a swirling but static volume of fluid. At the point of no flow, total work decreases significantly and power consumption is entirely related to overcoming friction.

The main difference between the two pumps is that a rotary blood pump can handle a low/no flow event very well by reducing its workload rather than increasing it like a displacement pump does. On this occasion, the pump’s behaviour is similar to a mixer: the impeller can contact, accelerate and do work only on the volume of fluid within the housing. During systole, the rotary pump work increases because of cardiac contraction with an increase in flow. The capacity of the pump is in excess of the physiologically supplied volumes and it can contact and accelerate more fluid. Therefore, more work is done and more power is required. Approximately 1.6 W are needed to pump 6 L/min at 120 mmHg. Power in excess of 1.6 W is wasted and converted into heat that must safely dissipate within the body [20].

In conclusion, rotary blood pumps are well suited for high-flow work with variable resistance. They are simple, durable and fundamentally different from volume displacement pumps in terms of hydrodynamics.

## 4. Rotary Blood Pumps: Axial or Centrifugal?

Although rotary blood pumps have changed the management of advanced heart failure patients, controversial issues remain whether an axial- or a centrifugal-flow design may be more suitable than the other in a clinical setting or whether a patient-specific approach should be considered according to the clinical condition [25,26,27].

The haemodynamic performance of axial- and centrifugal-flow rotary blood pumps is closely related to their pressure head and flow relationship (H-Q curve). Centrifugal-flow pumps have a flatter H-Q curve compared with axial-flow pumps. As a consequence, centrifugal-flow pumps are more efficient compared to axial-flow ones because of lower pressure head and friction losses resulting in lower power consumption. At the same time, a flatter H-Q curve means higher sensitivity to pressure head changes such as preload and afterload. Therefore, higher pressure head sensitivity in a centrifugal pump will lead to reduced ventricular suction events but lower flow rates in the presence of increased afterload. In view of their high pressure sensitivity, centrifugal-flow pumps show larger flow changes with greater flow pulsatility compared with an axial-flow pump as a result of the cyclical pressure head variation secondary to native ventricular contraction. Higher flow pulsatility gives larger variation in left ventricular end-systolic and end-diastolic volumes with higher aortic pressure pulsatility. Although, there are no differences in mean aortic pressure and flow between axial- and centrifugal-flow pumps for any given mean device flow rate at a constant preload or afterload [27]. A steeper H-Q curve in axial-flow pumps is related to a non linear current-to-flow relationship with inconsistency between measured and estimated device flow based on intrinsic pump parameters [28,29]. Finally, blood viscosity is another important parameter to take into account when comparing axial- and centrifugal-flow pumps [27].

Despite the higher preload and afterload sensitivity of centrifugal pumps, both devices show significantly lower sensitivity compared to the human heart [30]. At present, all continuous-flow pumps show reduced pressure and flow waveform pulsatility [31,32] without significant difference in end-organ perfusion and function [33,34,35]. Both axial- and centrifugal-flow pumps have their own specific features that may well be considered according to the clinical condition. The argument of a better unloading with an axial-flow pump [26] can be counteracted by systemic blood pressure control or increase in pump speed when a centrifugal-flow pump is used.

## 5. Ventricular Interdependence during Mechanical Circulatory Support

VADs operate in parallel with the native ventricles and affect both haemodynamic and mechanical interactions with changes in ventricular function [36,37,38]. Following LVAD insertion, potential alterations of right ventricular blood flow, compliance and anatomy can unmask an otherwise occult mild right ventricular dysfunction accounting for up to 30% incidence of right sided heart failure [39,40]. Right heart dysfunction has an impact on survival, length of stay in intensive care unit and hospital stay [41]. Early identification of patients who are at risk for right heart dysfunction may lead to concomitant temporary or long-term biventricular support at the time of LVAD implantation in order to reduce the negative impact on patient outcomes.

Long-standing left ventricular failure with mitral regurgitation leads to pulmonary hypertension, right ventricular hypertrophy and increased right ventricular stroke work index (*RVSWI*). LVAD insertion reduces pulmonary vascular resistance and increases venous return, which is compensated by the action of the trained right ventricle [42]. RVAD support will not be required. Chronic heart failure with high central venous pressure and low pulmonary artery pressure is highly suggestive of impaired right ventricular function [43]. A high central venous pressure reduces tissue perfusion and limits the rate of hepatic, renal, gastro-intestinal and cerebral recovery. RVAD support will be required following LVAD insertion.

Therefore, the presence of preoperative high pulmonary artery pressure is a favourable condition; high central venous pressure with tricuspid regurgitation, hepatomegaly and ascites is detrimental. Although LVAD insertion decreases left ventricular filling pressures and pulmonary vascular resistance with concomitant improvement in right ventricular function, earlier implantation before the onset of right heart failure leads to more favourable outcome [44]. The significant alteration of right ventricular loading conditions induced by a well-functioning LVAD in parallel with a weak left ventricle may have a potential beneficial and detrimental effect on the overall right ventricular function [36].

In view of the in-series nature of the right and left side of the heart, any increase in flow to the systemic circulation from the LVAD will result in an increased venous return to and an increased cardiac output from the right ventricle. Therefore, the right ventricle must be capable of increasing its cardiac output to at least the same amount of volume being pumped by the LVAD in order to achieve successful return of flow to the left heart and to the LVAD itself. In heart failure patients there is an approximate doubling of flow after the insertion of LVAD support compared to their preoperative status: from 1.6 ± 0.6 to 2.8 ± 0.5 L/min/m^2^ [45]. Therefore, the right ventricle must be capable of pumping at least 2.8 L/min/m^2^ in patients with isolated LVAD. In patients undergoing insertion of BiVAD support, the average LVAD flow increases from 1.4 ± 0.8 to 3.0 ± 0.5 L/min/m^2^ [45]. Therefore, the sum of blood flow from the RVAD and the output from the native right ventricle must be at least 3.0 L/min/m^2^ in patients with BiVAD support. Right ventricular dysfunction may only become clinically evident after LVAD support.

There is a consistent right ventricular response to left ventricular unloading by LVAD support in experimental settings: reduced right ventricular afterload, increased compliance and reduced contractility. Right ventricular contractility is impaired with a leftward septal shift but power output and myocardial efficiency are maintained through a decrease in right ventricular afterload and an increase in right ventricular preload [46,47,48,49,50]. In normal hearts, the overall effect is either no change or an increase in cardiac output. The response of the right ventricle is qualitatively the same in the presence of regional myocardial ischemia: reduced afterload, increased compliance and reduced contractility. However, anatomical ventricular interaction is accentuated with a greater reduction in right ventricular contractility. The global effect is either no change or a reduced cardiac output which may require inotropic or right ventricular mechanical support [51,52,53]. The response in human hearts is similar to the experimental setting: reduced right ventricular afterload, increased right ventricular preload and increased cardiac output in the absence of regional ischemia [54].

The in-series connection and ventricular interdependence can explain the observed right ventricular response to LVAD support [38]. The in-series connection decreases left ventricular preload, which will decrease pulmonary artery pressure. Right ventricular preload is increased by the increased left ventricular output during LVAD support and further assisted by the reduced left ventricular diastolic volume. The reduction in left ventricular diastolic volume increases right ventricular compliance leading to the observed changes in right ventricular shape and septal position with increased right ventricular volume and reduced right ventricular filling pressure. These effects have to compensate for the reduced left ventricular systolic assistance which leads to a reduced right ventricular systolic function. Because of ventricular interdependence, the reduced left ventricular systolic function decreases left ventricular assistance to right ventricular function leading to a positive feedback mechanism: the reduced left ventricular assistance decreases right ventricular systolic pressure and stroke volume followed by reduced left ventricular filling. The impaired right ventricular function reflects the observed changes in right ventricular septal position during systole and the reduction in right ventricular elastance and maximal rate of change of right ventricular pressure. Finally, the role played by the residual left ventricular wall stress must be taken into account despite the absence of left ventricular pressure due to decompression following LVAD support.

The possible mechanism for right ventricular failure during LVAD support can be compared to a bellows effect [55]. The right ventricle ejects blood through a uniform reduction in its free wall area and a reduced septal-to-free wall distance. Because of its large surface area-to-volume ratio, small decreases in septal-to-free wall distance cause large volume displacements [56]. Due to ventricular interdependence, the right ventricular free wall and the left ventricle contribute to right ventricular ejection during normal contraction [38]. Following LVAD support, right ventricular afterload is decreased in view of the increased motion of the right ventricular free wall while the left ventricular contribution is less because of decreased ventricular interdependence. In the presence of septal ischemia, the left ventricle moves away from the right ventricle. The right ventricular free wall cannot compensate leading to right ventricular impairment and reduced cardiac output. The key role is the change in the interventricular septum from its normal shape to a flattened or inverted shape in systole leading to a net motion of the septum away from the right ventricular cavity. Given the bellows-like contraction of the right ventricle, this septal motion significantly reduces right ventricular output and may explain the observed right ventricular impairment. The degree of right ventricular impairment is subject to underlying right ventricular dysfunction, the degree of right ventricular afterload reduction and the presence of regional ischemia, particularly in the septum.

In summary, increased venous return following LVAD support affects right ventricular function by increasing preload. At the same time, LVAD support improves right ventricular filling by unloading the left ventricle with reduction of its size and septal shift to the left. Right ventricular function is highly afterload dependent. LVAD support is beneficial for right ventricular function in relation to pulmonary vascular resistance [54]. LVAD support improves right ventricular function by reducing the afterload in patients with high pulmonary artery pressure secondary to left ventricular failure in the presence of a normal pulmonary vascular bed [47]. Complete decompression of the left ventricle leads to a significant reduction in left atrial pressure and concomitant decrease in pulmonary artery pressure followed by a decrease in right ventricular afterload. LVAD support will be detrimental in the presence of fixed high pulmonary artery pressure because of increased right ventricular afterload and volume secondary to high blood flow through an irreversible high pulmonary vascular resistance [36].

## 6. Vascular Pulsatility during LVAD Support

Pulsatility plays an important role in the human cardiovascular system. A clear understanding of this concept and its application in advanced heart failure during LVAD support is essential for appropriate clinical management.

Pulsatility is the magnitude of the arterial pressure pulse (*PP*), which is the difference between the maximum systolic ($AoP_{max})$ and minimum diastolic aortic pressure ($AoP_{min})$ as defined by the following equation [33]:

(19) $$PP=AoP_{max}-\;AoP_{min}$$

Another parameter used to quantify pulsatility based on flow is the pulsatility index (*PI*), which is the difference between maximum ($V_{max}$) and minimum ($V_{min}$) blood flow velocity related to the average blood flow velocity ($V_{mean}$) as defined by the following equation [33]:

(20) $$PI=\frac{V_{max}-\;V_{min}}{V_{mean}}$$

Continuous blood flow generated in a non physiological manner by rotary pumps avoids the need for valves and compliance chambers. The pulsatility index is a mode of operation of continuous flow VADs that mimics physiological flow and allows aortic valve opening during systole by adjusting the speed of the device [57]. Therefore, the pulsatility index is a measure of the size of flow pulse generated by the pump during a cardiac cycle. Maximum flow will occur during ventricular systole when the inlet-to-outlet pressure differential is the least, and minimum flow will occur during left ventricular diastolic filling when the inlet pressures are lower and the ΔP greater. Therefore, the *PI* can be reformulated as follows:

(21) $$PI=\frac{Q_{max}-\;Q_{min}}{Q_{avg}}$$

where $Q_{avg}$ is the average flow during the cardiac cycle.

Patients with very poor left ventricular systolic function have minimal pulsatility with $Q_{max}-\;Q_{min}=0$. The same low *PI* would be possible for a more functional left ventricle if the pump speed were excessive and the ventricle driven to collapse (speed excessive for preload).

The pulsatility index is the balance of native ventricular function and unloading by a continuous flow VAD. It is routinely monitored and adjusted to ensure safe automatic flow control and may be helpful when assessing a change in clinical status. Its value generally is set between 0.3 and 1.0 to ensure safe but responsive auto control [58].

When we consider the HeartMate II, the *PI* is the amount of pulsatility seen by the pump over a fifteen-second interval. This value is related to the amount of native heart function. *PI* is calculated from pump power normalized by mean power [22]. The pump recognizes only the speed (rpm) and the power required to maintain a certain speed. There is no flow detector. In the presence of native cardiac ejection, power usage increases and the flow is greater. The lower the *PI*, the greater the amount of support provided by the pump. The greater the *PI*, the more the native heart is ejecting. Therefore, the *PI* varies according to the hydration status, contractility, right ventricular function and exercise. Troubleshooting becomes more intuitive. If the controller shows decreased power consumption with a stable rotor speed, it means decreased work and flow. This may be related to decreased inflow due to volume depletion or cannula malposition or to increased afterload following vasoconstriction, hypertension or cannula geometry. Echocardiographic assessment can differentiate between poor drainage and poor inflow due to volume depletion. Increased power consumption with increased *PI* suggests more native cardiac ejection due to myocardial recovery or volume repletion from a dehydrated status.

Thrombus formation affects the reported flow estimate by changing the relationship between speed, power and flow. Thrombus can cause increased power consumption if the bearings require more friction work leading to an increased flow. However, thrombus can cause decreased power consumption if flow is reduced due to the presence of clot in the housing inflow or outflow. The worst scenario is when both effects balance and power remains roughly the same with an occult reduction in flow and clinical evidence of low perfusion with poor ventricular drainage on echocardiographic assessment.

Haemodynamic evaluation during LVAD support is critical in order to understand the interaction between the device and the vascular system. The pressure waveform undergoes significant amplification with discrepancy between central aortic pressure and the peripheral circulation. Pulse wave analysis can give helpful information about the pattern of pump ejection and the properties of the arterial system. The pressure waveform recorded at any site in the arterial tree consists of a forward waveform generated by the pump source (LV or LVAD) and a backward waveform, which is related to the incident wave reflected at peripheral sites. Analysis of the incident wave gives information about the pump source while analysis of the reflected wave gives information about the properties of the arterial system. Non-invasive measurement methods may prove particularly helpful for the evaluation of central haemodynamics in patients with heart failure and LVAD support [59,60].

Pulse pressure is inadequate for precise quantification of pulsatile and non pulsatile perfusion modes during chronic mechanical circulatory support because the generation of pulsatile flow depends on energy gradient rather than pressure gradient [61,62,63,64]. Arterial pressure and pump flow waveforms should be used to quantify different perfusion modes because different pulsatile pumps with the same pulse pressure have significantly different haemodynamic energy levels [65,66,67].

Energy equivalent pressure (*EEP*) and surplus haemodynamic energy (*SHE*) are instead appropriate to quantify pressure-flow waveform during acute and chronic mechanical circulatory support in a precise manner [33,61,65,68,69,70,71,72].

The energy equivalent pressure (*EEP*) is the ratio between the area under the haemodynamic power curve and the area under the pump flow curve during each pulse cycle:

(22) $$EEP\;\left(mmHg\right)=\frac{\int Q\cdot P\cdot dt}{\int Q\cdot dt}$$

where Q is the pump flow rate, P is the arterial pressure and dt is the increment in time.

Under adequate pulsatility, *EEP* is always higher than mean arterial pressure (*MAP*). The difference between *EEP* and *MAP* is the extra energy generated by each pulsatile or non pulsatile device. The difference between *EEP* and *MAP* in the normal human heart is approximately 10% [62]. In the presence of complete non pulsatile flow, *EEP* = *MAP* and the extra energy is zero. If both pressure and pump flow waveforms are available, then the energy equivalent pressure formula should be used [61], otherwise the use of pulse power index and/or pulsatility index is advisable [73,74].

The pulse power index (PPI) overcomes the limitations of the *EEP* formula, which requires both instantaneous arterial pressure and pump flow to be recorded: if one of the parameters is not available, the formula is not applicable. The PPI is the sum of the square of pump flow harmonics with the pump flow rate divided by the square of the mean flow:

(23) $$PPI=\sum_{i=0}^{n}\frac{A_{i}^{2}w^{2}i^{2}}{A_{0}^{2}}$$

where $A_{i}$ is the amplitude of the $i^{th}$ harmonic of flow, $A_{0}$ is the amplitude of mean flow and w is the frequency of flow (cycle/s). The pulse power index formula is useful in order to determine whether a continuous flow LVAD achieves physiological support [73,74,75].

The total haemodynamic energy (*THE*) is:

(24) $$THE\;\left(\frac{erg}{cm^{3}}\right)=1.332\;\frac{\int Q\cdot P\cdot dt}{\int Q\cdot dt}$$

where the constant value 1.332 is the converting factor from mmHg to dyne per cm^2^.

The surplus haemodynamic energy (*SHE*) is calculated by multiplying the difference between *EEP* and *MAP* by 1.332:

(25) $$SHE\;\left(\frac{erg}{cm^{3}}\right)=1.332\;\left(\frac{\int Q\cdot P\cdot dt}{\int Q\cdot dt}-\;MAP\right)$$

*SHE* is the extra energy that is generated only in the presence of some degree of pulsatility in pressure or flow. *SHE* is zero under complete non pulsatile conditions. At equal *MAP* and pump flow rate, adequate pulsatile flow always generates significantly more extra energy compared to non pulsatile flow [65,70,72]. This is a disadvantage when non pulsatile devices are used for either acute or chronic circulatory support. If we consider a 70 mL pneumatic pulsatile LVAD in a mock loop system, the *EEP* formula can be used to quantify the pressure-flow waveforms. At a constant pump flow rate of 5 L/min, aortic pressure of 80 mmHg, 90 mmHg and 100 mmHg, and pump rates of 65 bpm, 70 bpm and 80 bpm, the difference between *EEP* and aortic pressure is 9%–11% [68]. The extra haemodynamic energy generated by this device achieves the physiological level of 10%, which is equivalent to a normal heart. When axial flow or centrifugal pumps are used and the native heart is not ejecting, *EEP* becomes *MAP* and no extra energy is generated by these devices. As a matter of fact, most of the time in a clinical setting there is still some residual function of the native heart leading to some difference between *EEP* and *MAP*. In the presence of some myocardial recovery, it is possible to achieve near physiological pulsatility using a rotary blood pump [76]. The Jarvik 2000 is the only continuous flow device which generates complete non pulsatile flow under any condition [77], the remaining rotary blood pumps generate some degree of pulsatility [76].

## 7. Pulsatile or Continuous Flow?

Although rotary blood pumps have gained increasing acceptance for the management of advanced heart failure patients, there are substantial differences in arterial pulsatility during circulatory support with PF-LVADs and CF-LVADs in terms of vascular input impedance, energy equivalent pressure (*EEP*) and surplus haemodynamic energy (*SHE*). CF-LVADs reduce pulsatility from its physiological levels by significantly reducing *EEP* and *SHE* and increasing vascular impedance [31,73,74]. Prolonged diminished pulsatility may lead to vascular stiffening, which increases ventricular workload with reduction of myocardial perfusion because of early systolic aortic pressure wave reflections and attenuation of the baroreflex sensitivity [31].

The comparison of the results is more difficult to interpret when we consider haemodynamic changes and left ventricular unloading. A mock circulatory loop model with computer simulation [78] and a chronic ischaemic heart failure model [32] show greater left ventricular unloading with reduction in left ventricular end diastolic pressure and volume during support with CF-LVADs but also a disarrangement of the pressure-volume loop [78] and increased left ventricular systolic pressure and mean aortic pressure [32]. Other reports suggest no difference in left ventricular unloading but greater reduction in pulmonary artery pressure [79] or are in favour of PF-LVADs [80,81]. Interestingly, a more recent heart failure porcine model [82] shows higher left ventricular unloading with PF-LVADs with further confirmation of these results by a computational approach [83]. Overall, PF-LVADs show a higher rate of myocardial recovery compared to CF-LVADs [84,85], possibly related to the reduction in coronary blood flow [86,87] and cardiac atrophy in relation to the level and duration of support with CF-LVADs [88]. A recent review has found no significant difference in end organ perfusion or survival following transplantation in patients supported with CF-LVADs or PF-LVADs. The importance of intermittent aortic valve opening has been highlighted during both CF-LVADs and PF-LVADs support. The increased development of arterio-venous malformation and reduced Von Willebrand factor with CF-LVADs may contribute to higher incidence of gastro-intestinal bleeding. The incidence of right heart failure requiring right ventricular support is similar with both types of devices [89].

## 8. Control of Rotary Blood Pumps and Current Developments

A constant speed operation mode is the feature of all rotary blood pumps currently used in clinical practice. Although successful in unloading the heart, control on the cardiac workload is limited as shown by optimization studies of the interaction between rotary blood pumps and the cardiovascular system [90,91,92]. The ability to estimate pump flow and differential pressure is critical for the development of an automatic, physiological control system whose aim is to adapt pump output according to the haemodynamic changes that occur in an ambulant patient.

Computational modelling to evaluate a potential beneficial effect of pulsatile operation of CF-LVADs has played a significant role. The HeartMate II has been used to simulate cardiac assistance with constant speed and pulsatile mode [93] applying a computational approach based on left and right ventricular contraction with one-fibre model [94], systemic, pulmonary and coronary circulation based on lumped parameter models with heart rate regulation by a baroriflex model. The pulsatile mode has shown improved perfusion and unloading compared with constant speed offering more options to adjust the device settings according to the patient’s needs. Subsequently, different physiological control strategies for CF-LVADs have been proposed [95]. Numerical simulations of three support modes of CF-LVADs have been evaluated in a cardiovascular model in terms of blood assist index (*BAI*), left ventricular external work (*LVEW*), energy blood flow (*EBF*), pulsatility index (*PI*) and surplus haemodynamic energy (*SHE*). Continuous flow mode achieved the lowest *LVEW* with better LV unloading and promotion of myocardial reverse remodelling. Constant speed mode obtained the highest *EBF* with improved blood perfusion. Constant pressure head mode achieved the highest pressure pulsatility, which may be beneficial to maintain vascular function [96]. Ventricular interactions were not taken into account during these simulations. Speed modulation is being investigated as a potential solution to optimize control of pulsatility and cardiac workload [97,98]. In addition, a preload-based Starling-like controller is being developed and proposed as an alternative to pulsatility control. Numerical simulations seem to show superiority of a Starling-like controller in comparison with pulsatility control and constant speed operation during transition from baseline to exercise, blood loss and reduced left ventricular function [99]. The theoretical background consists of a linear controller for pump flow which mimics the response of the native left ventricle to changes in preload and is obtained using pump flow pulsatility as the feedback variable. The controller can adapt to accommodate longer-term changes in cardiovascular parameters [100].

## 9. The Role of Computational Fluid Dynamics in Thrombosis Modelling and Optimization

**Figure 1:**
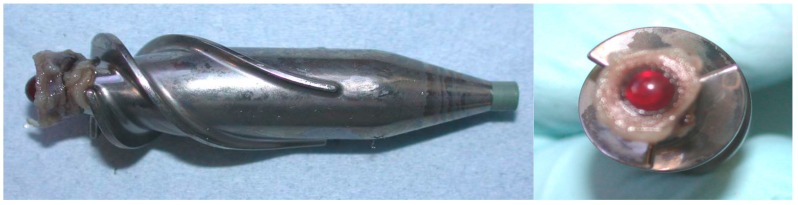
HeartMate II impeller: note thrombus formation around the ruby inlet bearing.

Despite the sophisticated level of technology currently available, pump thrombosis remains the most feared complication with potential disastrous consequences and impact on clinical outcome. Elevated flow stresses in the non physiological geometries of blood re-circulating devices are closely related to thrombus formation by chronically activation of blood platelets (Figure 1). This, rather than haemolysis, seems the key aspect of blood trauma in these devices [101]. VADs operate in a flow regime which has been difficult to simulate: the transitional region at the boundary of laminar and turbulent flow. Different approaches have been used but controversial issues still remain. Computational Fluid Dynamics (CFD) is an investigational tool that has played a significant role in the design and development of VADs [102], modelling of thrombus formation and haemolysis [103,104] and patient-specific fluid-structure interaction analysis [105].

The presence of non physiological flow patterns enhancing the haemostatic response is one of the major issues in blood recirculating devices. The activation and sensitization response of platelets in blood recirculating devices is dependent on shear loading rate, shear stress magnitude and exposure time [106]. The interaction between flow-induced stresses and blood components is related to the hypothesis that thromboembolism in blood recirculating devices is initiated and maintained by the non physiological flow patterns and stresses which activate and enhance platelet aggregation [107]. A reliable method capable of quantitatively accurate predictions of flow-induced blood haemostatic activation is essential in order to reduce thromboembolism in cardiovascular devices. Device thrombogenicity emulation (DTE) methodology is a recently developed optimization strategy aimed at the reduction of the thrombogenicity potential in blood recirculating devices with particular reference to ventricular assist devices [108]. DTE combines in silico advanced numerical simulations with in vitro techniques by correlating device haemodynamics with coagulation markers. The optimization process begins with in silico modelling first followed by experimental emulation of the device specific stress loading waveforms in a Haemodynamic Shearing Device [109] where device specific effects on platelet activation are measured with a modified prothrombinase assay [110]. Flow through a ventricular assist device is modelled with a high fidelity two-phase fluid-structure interaction (FSI) simulation resolving all components of the stress tensor relevant to flow-induced thrombogenicity. The cumulative stress loading history that may drive platelets beyond their activation threshold is calculated along multiple flow trajectories with the combined effect of shear stress and exposure time [111,112,113]. The stress tensor is extracted from the simulations along the corresponding platelet trajectories and rendered into a scalar stress value [114,115] so that both viscous and turbulent stresses ($\tau_{ij}$) are considered in the formulation of stress accumulation:

(26) $$\sigma =\frac{1}{\sqrt{3}}\;\sqrt{\tau_{11}^{2}+\;\tau_{22}^{2}+\;\tau_{33}^{2}-\;\tau_{11}\cdot \;\tau_{22}-\;\tau_{11}\;\cdot \;\tau_{33}-\;\tau_{22}\;\cdot \;\tau_{33}+\;3\;\left(\tau_{12}^{2}+\;\tau_{23}^{2}+\;\tau_{13}^{2}\right)}$$

A linear product of instantaneous values of the stress, σ, and exposure time, $t_{exp}$, is assumed for the stress accumulation (SA) and a summation is obtained as follows:

(27) $$SA=\sigma \;\cdot \;t_{exp}=\int_{t_{0}}^{t_{exp}}\sigma \left(t\right)dt\approx \sum_{i=1}^{N}\sigma_{i}\;\cdot \;\Delta t$$

where $\sigma_{i}$ is the nodal scalar value extracted from the total stress tensor described above, Δt is the corresponding time step between successive nodal points and *SA* is given in Pa·s.

Although the argument of other stress accumulation models is in favour of various power law combinations in the following form:

(28) $$\sum \sigma^{a}\;\cdot \;\Delta t^{b}$$

the previously proposed model seems to better predict platelet stress accumulation in blood recirculation devices [111,115,116]. The probability density function of stress accumulation along multiple trajectories is used as a surrogate for the evaluation of the overall thrombogenic potential of different ventricular assist devices (VADs); in other words, a sort of thrombogenic footprint of VAD design including specific regions of interest such as inlet flow straightener (vanes), inlet hub and bearing taper, impeller, impeller-shroud near wall region and tip-shroud gap clearance.

## 10. The Impact of Acquired von Willebrand Syndrome in VAD-Patients

Patients undergoing CF-LVADs insertion show features suggestive of acquired von Willebrand disease caused by deficiency or dysfunction of von Willebrand Factor (vWF), a key clotting protein whose interaction with platelets and vessel wall generates primary haemostasis. The development of high shear stresses related to pump design and resultant non physiological flow pattern of axial and centrifugal devices seem to play a major role for the occurrence of this syndrome leading to destruction of vWF with subsequent bleeding and thromboembolic events [117,118]. Although the mechanistic pathways of vWF degradation remain unclear, multiple mechanisms are probably responsible including vWF cleavage by ADAMTS-13 metalloproteinase with mechanical demolition being the likely dominant process [119]. The preliminary results of the MOMENTUM 3 trial have shown favourable outcomes for the HM III with no pump thrombosis at 6 months [19,120] without loss of high molecular weight vWF multimers to the same extent caused by the HM II [121]. These findings seem even more encouraging than a previous comparison between HM II and HW HVAD [122], which may well be related to differences in design between HW HVAD and HM III. The breakdown of vWF remains a shear stress related phenomenon while speed manipulation does not decrease vWF degradation [123]. From a computational point of view, continuum (top-down) and multiscale (bottom-up) methods have been proposed, each with advantages and disadvantages. An integrated, computationally efficient, robust and simple enough coagulation model may well benefit from a combination of both methods and complemented with a mesoscopic-like approach for the modelling of particle interactions such as fibrin polymerization and platelet activation [124].

## 11. Discussion

**Figure 2:**
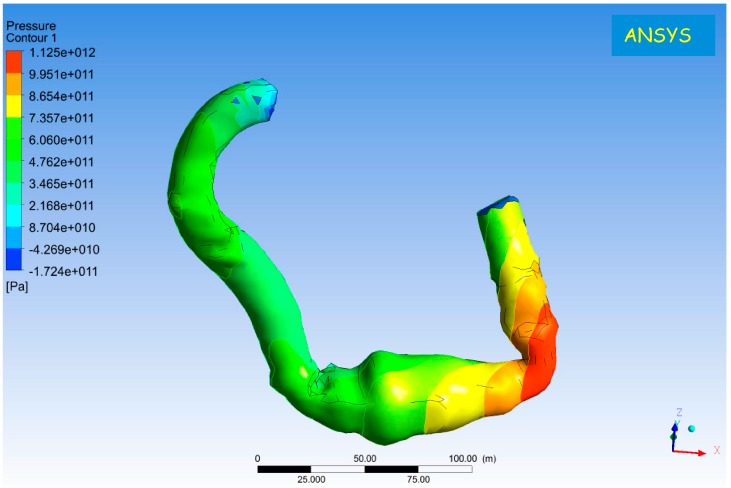
3D reconstruction from CT-scan of HeartMate II following prolonged circulatory support. Simulation with k-ω SST (shear stress transport) model: mass flow inlet, 0.1 kg/s; pressure outlet, 100 mmHg; rotational speed ω = 9200 rpm.

Although cardiac transplantation is repeatedly considered as the “gold standard” for the treatment of advanced heart failure, its epidemiological impact remains low and a luxury for a limited group of patients such as those with palliated complex congenital heart disease or dilated cardiomyopathy with significantly impaired right ventricular function [125,126]. Prolonged mechanical circulatory support with rotary blood pumps has the potential to largely overcome heart transplantation and provide an unrestricted “off the shelf” solution in terms of symptomatic relief and improvement of quality of life for those patients without access to transplantation [125,126,127]. In view of the increasing and unnecessary complexity of the donor allocation process, the insertion of a left ventricular assist device may well become the primary treatment for all suitable patients in advanced heart failure [128]. The preliminary results of the ROADMAP trial have already shown a beneficial effect in INTERMACS 4–6 patients while the REVIVE-IT, designed to evaluate the role of LVADs in INTERMACS 7 patients, is currently not recruiting in view of the initial controversy related to its protocol. The concern remains about the timing of such a trial and whether the current technology is ready for it despite the fact that the evolution from pulsatile- to continuous-flow devices has made mechanical circulatory support a viable option for patients in advanced heart failure. The Berlin experience review [129] has shown the clear feasibility and need for this approach in order to face the increasing patient population. At the same time, the review has highlighted the issues that still need to be addressed in order to achieve even wider acceptance. All of us have enjoyed watching Lee Majors and Lindsay Wagner in “The six million dollar man” and “The bionic woman” in what was considered science fiction at that time. Not anymore! Prosthetic limbs have gone a long way making sport competition a reality. We are witnessing a complete technological revolution. Whether this is a good or bad thing remains a matter of debate. The problem is that heart failure needs addressing in a serious way. Rotary blood pumps may well be the way forward. Speed modulation and a more physiological control are addressing the vascular complications related to non pulsatility during prolonged support. Despite the initial limitations of induction-based transcutaneous energy transmission systems (TETS) with restrictions on separation distance and alignment between transmitting and receiving coils, a free-range resonant electrical energy delivery system (FREE-D) may well become the most suitable approach to a totally implantable LVAD that charges its internal batteries wirelessly with potential for complete mobility and free-infection risk [130,131]. A pre-operative strategy to decide suitability for a device would be an attractive option, which may well happen in a non distant future. Patient-specific modelling may become a daily approach for clinical management and training of medical and nursing staff (Figure 2). All of this is available but requires willingness to change attitude towards a different type of approach, which may become the key to successful outcome and the beginning of a complete revolution in patient care. No magic bullets, only different way of thinking. We must be able to provide true quality of life and give a valid alternative to palliative care. In summary, CF-LVADs based on speed modulation with more physiological control and powered wirelessly should be considered the next achievable target in the short-term.

## 12. Conclusions

Rotary blood pumps are here to stay. Their features have proved that long-term mechanical circulatory support is a feasible option although the absence of pulsatility has been associated to peculiar complications, which are currently being addressed. There are now enough pumps on the market: it is time to focus on the complications in order to achieve the full potential and selling-point of this type of technology for the treatment of the increasing heart failure patient population.

## Abbreviations

The following abbreviations are used in this manuscript:

REMATCH	Randomized Evaluation of Mechanical Assistance for the Treatment of Congestive Heart Failure
ROADMAP	Risk Assessment and Comparative Effectiveness of Left Ventricular Assist Device (LVAD) and Medical Management in Ambulatory Heart Failure Patients
REVIVE-IT	Randomized Evaluation of VAD Intervention before Inotropic Therapy
TETS	Transcutaneous Energy Transfer System
LVAD	Left Ventricular Assist Device
RVAD	Right Ventricular Assist Device
